# Anorectal balloon cell melanoma: a rare variant

**DOI:** 10.4322/acr.2023.459

**Published:** 2023-12-15

**Authors:** Priyanka Sameer, Pallavi Srivastava, Saumya Shukla, Nuzhat Husain

**Affiliations:** 1 Institute of Medical Sciences Dr. Ram Manohar Lohia, Department of Pathology, Lucknow, Uttar Pradesh, India

**Keywords:** Rectal neoplasms, Immunohistochemistry, Abdomen, Balloon Cell Melanoma

## Abstract

Balloon cell melanoma is a rare presentation of malignant melanoma, usually on the skin, with less than 100 cases reported. Mucosal BCM is even rarer, with only one case of anorectal BCM reported in English literature. The diagnosis is based on the histopathologic findings of a tumor composed of large, foamy melanocytes, with or without pigmentation, and confirmed by immunohistochemical studies showing expression for melanocytic markers. The foam cell appearance of the tumor cells and the lack of melanin pigment lead to a diagnostic dilemma, mostly when presented at an unusual location. Herein, we report a case of balloon cell melanoma at the anorectal junction in a 73-year-old male patient complaining of constipation and bleeding per rectum. Surgical resection was performed with no evidence of recurrence after three years of close follow-up. We believe this case will raise awareness among the medical community to consider this tumor a differential diagnosis in rectal masses.

## INTRODUCTION

Anorectal malignant melanoma (ARMM) is a malignant neoplasm,^1^ accounting for 1.3% of all melanoma and 16.5% of all mucosal melanoma.^2^ ARMM affects the anorectal region, mostly located 6 cm from the anal rim.^3^ The English medical literature counts on one case report of anal melanoma of the balloon cell variant. ARMM presents with anal bleeding, pain, and pruritis, and endoscopic findings mimicking a benign polypoid mass. Hence, these non-specific data lead to the probability of under-diagnosis. On radiology and histopathology, the sheet of non-pigmented balloon cells may imitate a benign lesion, including xanthomatous lesions, mucopolysaccharidoses, or histiocytic lesions. The main therapeutic modality is surgical resection; however, further studies are necessary to establish a more precise roadmap, including immunotherapy or other targeted therapies. The cutaneous site balloon cell melanoma has shown a metastatic potential to various sites^4^, including the inguinal region, legs, neck, brain tissue, the scar of the primary tumor, thorax, and abdomen. The mucosal variant of balloon cell melanoma should be reported in view of high metastatic potential. We report a rare case of balloon cell melanoma (BCM) in a 73-year-old male presenting as a polypoid pedunculated mass on the anterior wall of the anal canal. Abdominoperineal resection was performed.

## CASE REPORT

**Figure 1:**
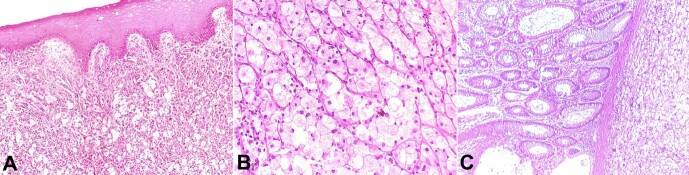
Photomicrograph of the polypoid lesion showing in **A** and **B** – unremarkable anal mucosa with underlying tumor disposed in sheets of epithelioid and balloon cells having abundant eosinophilic to vacuolated cytoplasm with central to eccentric hyperchromatic nucleoli; **C** – shows overlying rectal mucosa with subepithelial zone infiltrated by tumor. (H&E, **A** – 100X, **B** – 200X, **C** – 100x)

A 73-year-old man, a chronic smoker without previous comorbidities, presented with constipation for 4 months. There was a history of hemorrhoids with mild per-rectal bleeding treated by a local practitioner. The rectal examination identified a polypoidal lesion with a stalk on the wall of the rectum longitudinally, approximately 2 cm above the anal verge. Perianal skin was normal. The abdominal ultrasonography (USG) showed a polypoidal mass of 20 mm along the rectum. The colonoscopy confirmed the presence of a 2.0 cm polypoid mass in the distal rectum with a sloughed base and easy bleeding on touch. MRI pelvis revealed a well-defined lobulated polypoidal mass measuring 43 x 28 x 47 mm on the anterior wall of the distal rectum with involvement of anorectal junction, 25 mm from the anal verge. No lymphadenopathy was seen. Routine complete blood count, renal, and liver function tests were within normal limits. The carcinoembryonic antigen level was 5.0 ng/ml (0.3-2.7 ng/ml). The biopsy rendered the diagnosis of an undifferentiated tumor. The patient was admitted to the Surgery Department and underwent abdominoperineal resection (APR) under general anesthesia. We received an APR specimen. On gross examination, the surgical specimen showed a 3.8 x 3.5 x 2.5 cm polypoidal pedunculated mass at the anorectal junction on the anterior wall. The mass cut surface was homogenous, and the adjacent mucosa was unremarkable. Eight lymph nodes and the resection margins were free of tumor. The histological examination revealed the anorectal mucosa with an underlying tumor disposed in sheets and islands of foam cells with interspersed focally pigmented epithelioid to spindle cells with prominent nucleoli (Figure 1A). The foam (balloon) cells were large with abundant vacuolated cytoplasm, central to eccentric hyperchromatic nuclei, and inconspicuous nucleoli (Figure 1B).

**Figure 2:**
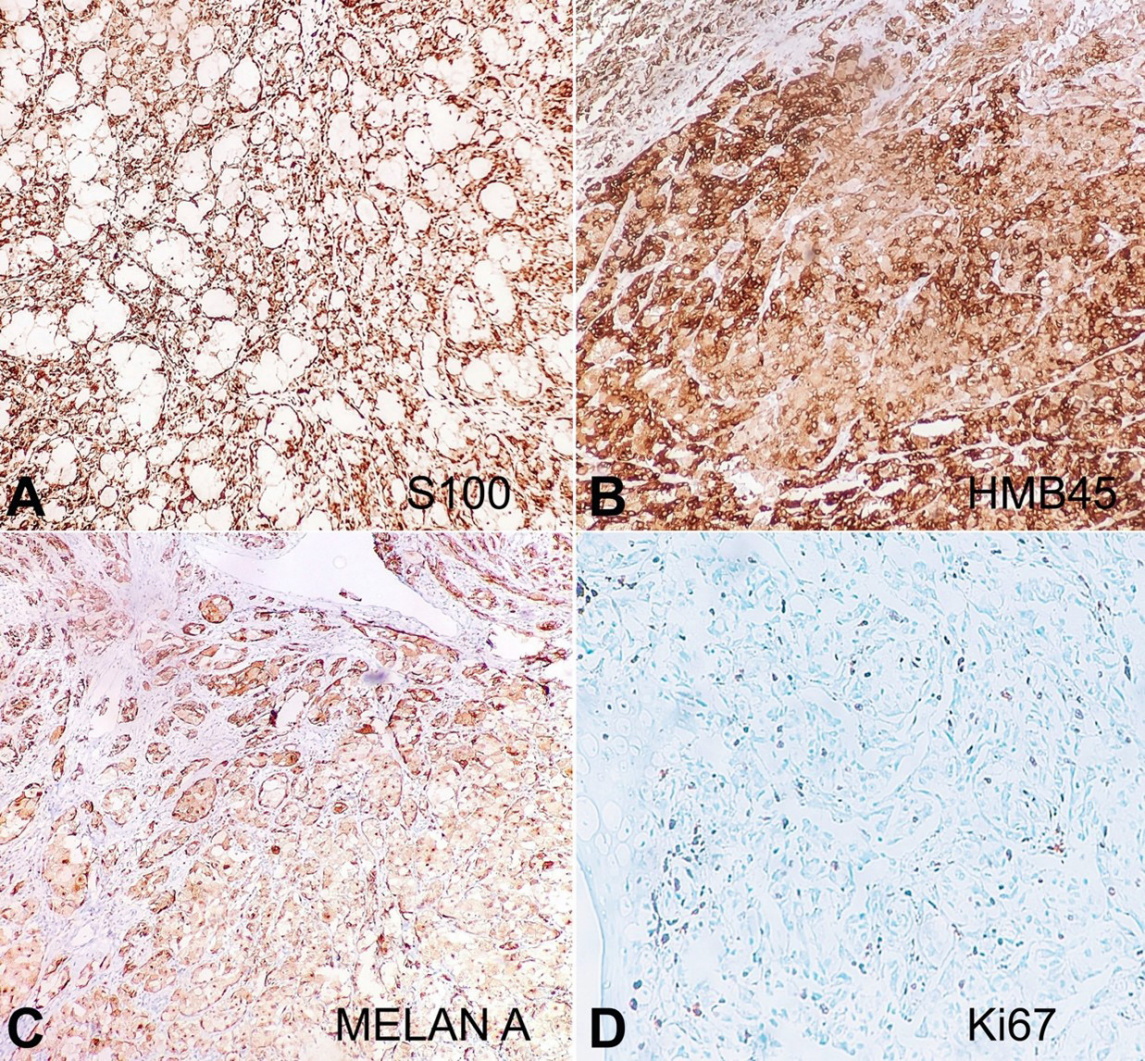
Immunohistochemical findings: **A** – Tumor showing diffuse expression for S-100, **B** – HMB-45, **C** – Melan A, negative expression for **D** – Ki 67 proliferation index. (Original magnification, **A-D** DAB 200x)

Frequent mitoses, including atypical forms, were seen. The balloon cells and pigmented epithelioid cells showed positive expression for S-100 protein (Figure 2A), HMB-45 (Figure 2B), Melan-A (Figure 2C), and negative expression for pan-cytokeratin and epithelial membrane antigen (EMA). Staining with Ki-67 was positive in almost 40-50% of the tumor cells (Figure 2D). Tumor cells also showed negative expression for PDL-1 (VENTANA SP263) and BRAF V600VE (VE1) mouse monoclonal antibody.

The diagnosis of the Malignant Melanoma-balloon cell variant was rendered. The postoperative condition was uneventful, and the patient was disease-free after 3 years of close follow-up.

## DISCUSSION

BCM is a rare variant of malignant melanoma of the anal canal, with only one case reported in the English literature. Gardner and Vazquez^5^ reported the first case of BCM with most cases of cutaneous origin, sporadic cases of non-cutaneous origin have been reported, including the anal canal,^6^ ciliary body,^7^ urethra,^8^ temporoparietal region,^9^ and choroid plexus^10^ (Table 1). We searched the PubMed, Medline, and Embase databases using the search terms malignant melanoma anorectum, balloon cell melanoma non-cutaneous, and cutaneous sites updated until 1979 and limited to English language papers. The Medical Subject Headings and keywords used for the search were “malignant melanoma anorectum”, “cutaneous balloon cell melanoma”, “noncutaneous balloon cell melanoma”, and “case reports”.

**Table 1:** Review of non-cutaneous balloon cell melanoma

**Ref**	**Age/ gender**	**Site**	**Follow up**	**Treatment**
9	30/F	Right temporoparietal region	NA	NA
10	59/M	Choroid plexus	Metastasis to lung, bone	Surgery
	75/F	Choroid plexus	Metastasis to Liver	
6	73/M	Anal canal	Recurrence	NA
8	76/F	Urethra	No evidence of recurrence or metastatic disease	Extensive surgical resection (anterior exenterative surgery and vaginectomy)
7	60/F	Ciliary body	No evidence of recurrence or metastatic disease	Surgery (enucleation)
CC	73/M	Anorectum	No evidence of recurrence or metastatic disease	

NA=Not available; CC= current case; Ref= reference.

The histopathological analysis of BCM revealed the proliferation of tumor cells disposed of in diffuse sheets, nests, or cords comprising at least 50% of the tumor population. The foamy tumor cells mimic sheets of foamy histiocytes, which may be a pitfall for an unaware pathologist on this rare variant in anorectal location. The pathogenesis of ballooning morphology is not clearly understood; however, aberrancy in melanosome maturation is attributed to this change rather than a degenerative process.^11^ The electron microscopy findings revealed dilatation and emergence of melanosomes, leading to ballooning of the cells.^12^ Due to the unusual site, foamy cell appearance, and lack of melanin, the differential diagnosis should include (i) signet ring cell carcinoma, (ii) perivascular epithelioid cell tumor, (iii) lipo-sarcoma, (iv) gastrointestinal stromal tumors, (v) neuroendocrine tumor or (vi) epithelioid leiomyosarcoma. Metastasis from renal clear cell carcinoma should also be considered in case of amelanotic lesion, and metastatic melanoma should be ruled out by thorough and detailed clinical and radiologic evaluation, including ophthalmologic examination for uveal melanoma. Immunohistochemical reactions using monoclonal antibodies for S100 (more sensitive), HMB45 (more specific), Melan-A, and SOX10 are used in the diagnosis of primary and metastatic melanoma (97%–100% sensitivity)^13 The prognosis of non-cutaneous balloon cell melanoma is not well defined due to the limited number of cases reported; however, a five-year survival rate of <20% has been documented in malignant melanoma of the anorectal region.14 APR is the best treatment for locoregional disease. Consensus on the therapeutic option is not defined due to the rarity of this variant; however, advanced malignant melanoma has shown promising results with targeted therapeutic options, including immunotherapy (CTLA-4 inhibitors, PD-1/PD-L1 inhibitors or IFN/IL-2), antiangiogenic therapy (Bevacizumab, endostatin) or targeted therapy (BRAF/MEK inhibitors, KIT targeted therapy, PI3K-AKT target therapy or mTOR inhibitors). IHC for PD-L1 and BRAFV600E was performed in the current case with a focus on targeted therapy; however, the balloon cells showed negative expression for both markers, and a single case finding may not reflect the actual receptor status in this variant. Further studies of the variant and its IHC characterization should be performed to understand the biology and pathogenesis, focusing on immune checkpoint inhibitors and other targeted therapies.15^

## CONCLUSION

The ARMM is considered similar to mucosal melanoma due to similar clinical, genetic, and biological characteristics. Pathologists should be aware of the rare variant and differential diagnosis of BCM; hence more cases need to be documented for a better understanding of the pathogenesis, prognostic, and predictive factors.
